# New Out-Patient Department at Charing Cross Hospital

**Published:** 1904-10-22

**Authors:** 


### NEW OUT-PATIENT DEPARTMENT AT CHARING CROSS HOSPITAL.

The new out-patient department at Charing Cross Hospital, which was opened on October 1st, comprises a large waiting hall, dispensary hall, medical and surgical rooms, dressing-rooms, etc., night accident wards, $x$ ray and Finsen lamp rooms, and electrical and medical baths. The entrance is in King William Street, and there are two casualty rooms, medical and surgical, with separate lavatory arrangements for men and women. The sinks are fitted with a Berkfeld steriliser and filter, a thermometer being supplied to register the required temperature. Each sink is fitted with elbow taps and an anti-splash nozzle, and cold, tepid, and hot water can be obtained by single movements of the taps. These fittings are by Messrs. Doulton, and are similar to those recently supplied to the British Hospital at Constantinople. Ordinary out-patients—other than casualties—have a separate entrance, and wooden benches are ranged along the corridor, which has a gate to prevent overcrowding to the waiting hall. The new George Stagg Ward, open for accidents from 9 P.M. to 7 A.M., is, like the two casualty rooms, tiled in white and green. The linoleum with which the floors of the two rooms into which the ward is divided are covered is laid so as to avoid corners, the edges being turned up to meet the tiles. There are two beds for men and two for women. Passing a small duty-room, with hot and cold water, lavatory, etc., the beautifully tiled waiting hall is reached. The walls are white, with a green ornamental border running round, above which, as a frieze, is a series of pictures of the chase, in coloured tiles. There are two drinking fountains fixed in the wall, with cups attached; a provision of which we cannot approve, for reasons which must be obvious to all.

The four consulting-rooms, with their dressing-rooms and separate doors for egress through the dispensary hall to Chandos Street, are devoted to surgical, medical, light, throat and ear, and skin treatment, the patients being first interviewed by the resident medical officer in a room set apart for the purpose, and by a clerk who inquires into the suitability of the case. There are separate entrances for women and men to the waiting-hall. Separate rooms are set apart for minor gynæcological operations, adenoids, etc. Besides electrical baths, including a "Sunbeam," there are six medical baths, separated by tiled partitions. Considerable space is devoted to the electrical department, which contains a Finsen lamp of the latest pattern.

The dispensary waiting-hall, from which the consulting-rooms open out, is a handsome apartment with Doulton columns and a teak floor.

The electrical department, baths, etc., are on the sub-basement, and the whole of the new premises is necessarily lighted from the roof by means of skylights. Electric light is fitted throughout. The passages are tesselated and the heating is supplied by means of coils.

